# Characterisation of Luvisols Based on Wide-Scale Biological Properties in a Long-Term Organic Matter Experiment

**DOI:** 10.3390/biology12070909

**Published:** 2023-06-25

**Authors:** Zsolt Kotroczó, István Fekete, Katalin Juhos, Nándor Prettl, Priyo Adi Nugroho, Gábor Várbíró, Borbála Biró, Tamás Kocsis

**Affiliations:** 1 Department of Agro-Environmental Studies, Hungarian University of Agriculture and Life Sciences, H-1118 Budapest, Hungary; juhos.katalin@uni-mate.hu (K.J.); nandor.prettl@gmail.com (N.P.); priyo.nugroho@puslitkaret.co.id (P.A.N.); biro.borbala@gmail.com (B.B.); 2 Institute of Environmental Science, University of Nyíregyháza, H-4400 Nyíregyháza, Hungary; feketeistani@gmail.com; 3 Department of Tisza River Research, Danube Research, Institute, Centre for Ecology of HAS, H-4026 Debrecen, Hungary; varbiro.gabor@ecolres.hu; 4 Department of Food Microbiology, Hygiene, and Safety, Hungarian University of Agriculture and Life Sciences, H-1118 Budapest, Hungary; kocsis.tamas.jozsef@uni-mate.hu

**Keywords:** soil proteins, MALDI-TOF, organic matter, soil biology, dehydrogenase activity DIRT

## Abstract

**Simple Summary:**

Global climate change has a significant impact on soil decomposition processes through the alteration of temperature and precipitation and, in connection with them, through changes in the quantity and quality of biomass production in ecosystems. The role of soil organic matter (SOM) is particularly important, as the consequences might rapidly affect soil carbon stocks. These processes have a global impact on the CO_2_ content of the atmosphere and a local impact on the fertility of soils. In our research, which is based on a transcontinental litter manipulation, detritus input, and removal treatment (DIRT) project, we investigated how quantitative and qualitative changes in litter inputs can affect decomposition processes and carbon storage capacity of soils in relation to SOM content. The main question of the study is how various litter treatment sites respond to additional and/or removed organic matters through assessed soil-biological parameters. The changes were monitored with some potential soil-biological indicators, such as through examining enzyme activity, CO_2_ emissions, and labile carbon stocks (POXC) used by organisms in performing decomposition processes. The model experiment provided a great background highlighting organic matter’s importance in soil-biological processes and soil ecosystem functioning.

**Abstract:**

Soil organic matter is a biological system that functions as an integrated whole. These assemblies have different properties, functions, and decomposition times. SOM is one of the main determinants of soil productivity. Our studies were carried out in a temperate deciduous oak forest on Luvisols soil. In the DIRT Project (Detritus Input and Removal Treatments), the following treatments were applied: Double Litter, Double Wood, Control, No Litter, No Root and No Input. Our objective was to compare the effect of withdrawal or doubling of organic matter on the protein pattern of the soil and the biological activity and changes in labile C (permanganate-oxidizable carbon) content in a long-term organic matter manipulation experiment. Patterns of thermostable proteins, soil dehydrogenase enzyme activity, CO_2_ emission, and POXC content were measured at the most biologically active soil depth of 0–5 cm after 23 years of treatment. Our results show that the enzyme activities of the litter removal treatments were significantly reduced compared to the doubling treatments, as were the values of soil respiration. The same significant difference was also detected in the C content of the soils of the treatments. Based on cluster analysis of the protein profile of the soil samples, the No Litter and No Input treatments were significantly different from the other treatments. This shows that specific organic matter is needed to enhance soil biological activity and the associated POXC content.

## 1. Introduction

Many studies and models have already been prepared on the effect of climate on vegetation, but we know less about how these changes affect the soil’s organic matter stock. The main reason for this is that organic matter content is more difficult to determine than plant biomass in wide geographical conditions. According to future scenarios of global warming, changes in temperature and precipitation distributions are expected to affect primary production, the diversity of living organisms, and the functioning of the ecosystem [1,2]. All these factors will affect the evolution of the quality and quantity of organic matter. They determine its entry into the soil, the intensity of the nutrient cycle, and the change in its dynamics [3,4]. Organic matter in the soil can be considered as a biological system functioning as an integrated whole [5]. At the same time, there are components of organic matter that can be treated as separate units. These stocks have different properties and decomposition times, and their quantity and proportion vary depending on the soil, one of the most important determinants of which is soil use and the input of organic matter into the soil [6]. The influence of temperature and precipitation on soil C dynamics has been reported in several studies [7,8]. However, these results are varied, as climate has both direct effects on the soil C balance through biological processes as well as indirect effects through changes in vegetation, the rhizosphere effect, and the input of organic matter. Several studies report that climate change has complex effects on the carbon balance of forest ecosystems [9,10]. Temperate deciduous forests in the northern mid-latitudes play an important role in the global C cycle and accumulation [11] and are thus significant in terms of their climate-modifying and mitigation effects [12].

Considering climatic and microclimatic effects on soil carbon turnover is also important, as they significantly influence microbial activity and, through this, the decomposition process and thereby the amount of carbon stored in the soil. During SOC studies conducted at the Síkfőkút DIRT site, we observed a significant decrease in the plots with live root removal treatments (NR, NI), in which significantly wetter soils, caused by the lack of evapotranspiration, played an important role [13]. These impacts include responses to rising atmospheric CO_2_ levels and climate change. Carbon stocks and their accumulation in forests are also strongly influenced by changes in land cover and land use. However, forest ecosystems in many parts of the world are expected to become drier as a result of climate change; evapotranspiration will increase with warming, drought periods will become more frequent and longer, and extreme weather periods during the growing season will become more frequent [14]. Dry summers, previously typical of Mediterranean climates, are projected to become even more frequent across much of central and parts of western Europe, reshaping the ecosystems we know today [15,16]. Together, these processes determine the carbon turnover of soils, that is, which part of the organic matter input goes into the soil’s “permanent” carbon stores, and which part is used by soil microorganisms during the decomposition–transformation processes. This is proven via the differences observed between international DIRT project sites. The litter treatments used in the DIRT project caused significant differences in the soil carbon balance [17]. At least as interesting is the difference experienced between individual DIRT sites, which shows the different extent of the priming effect. These processes caused significant differences in the soil carbon stock of the treatments for sites with different climatic conditions [18]. In the case of the driest Síkfőkút DIRT site, the priming effect appeared in a much smaller extent than in the case of the other DIRT sites. Since soil organic matter does not have a precisely defined chemical composition, soil organic carbon (the dominant elemental component of SOM) is usually measured and used more often in scientific research. Small changes in the total organic matter content of soil are difficult to detect due to the typically higher reserves and the diversity of soils.

Therefore, in the 1970s, several systems were developed to further subdivide the total organic carbon content based on different principles. Various proposals were made to standardize the potassium permanganate oxidation of soil carbon [19,20]. The aim of these measurement methods is to determine the “active”/”labile” (more available to plants and microbes) carbon content of the soil, which includes the carbon stored in the soil microbial biomass, organic matter, and carbohydrate molecules. The resulting labile (POXC) organic carbon content is more sensitive to soil amendments than the total organic carbon content.

Understudied proteins of different origins are also part of soil organic matter [21,22,23]. Proteins are macromolecules consisting of peptides and chains of amino acids. Their biological role is well characterized by the fact that they are present in all living cells and are a defining part of soil’s organic matter stock. According to their function, they can be enzymes, transport proteins, protective proteins, toxins, hormones, structural proteins, motor proteins, and reserve proteins [24]. In terms of their origin, they can come from many sources; both plant and microbial biomass can contain them. However, proteins produced during microbial activity are the most significant. Essential soil proteins are glomalin-related soil proteins (GRSP) produced by AMF fungi, which are proportional to the amount of mycorrhizal fungi found in the soil. In this connection, much of the literature mentions that GRSP is a good indicator of soil health and use [25], but its amount also depends on environmental factors and soil condition. Several studies have shown that glomalin also affects soil structure as it stabilizes soil aggregates containing fine particles as a glue and plays an essential role in soil fertility [26,27]. As a glycoprotein, glomalin stores carbon in the form of proteins and carbohydrates. GRSP released by AMF is an important component of soil organic carbon (SOC) pools [27]. C from mycorrhizae accumulates in soil through extraradical hyphae as a pool for C storage, where the contribution of mycorrhizae to accumulated organic C in the soil ecosystem is about 54–900 kg ha^−1^ [23,26].

Soil enzymes also play an essential role in the mineralization of nutrients and the decomposition of organic matter, and their function is a key factor in the supply of nutrients to plants [28]. Dehydrogenase activity (DHA) is used to estimate total microbial activity [29]. Many environmental factors, including soil moisture, oxygen availability, oxidation-reduction potential, pH, organic matter content, soil profile depth, temperature, etc. significantly affects the level of DHA in the soil [28].

This work aimed to investigate the effects of long-term organic matter treatments in a *Quercetum petraeae-cerris* forest association. (i) We were looking for the answer to what effect the long-term withdrawal or doubling of organic matter has on the microbial activity of soil, as well as (ii) how these organic matter treatments affect the permanganate-oxidizable carbon content of the soil. Another goal of our work was to (iii) compare the protein profile of the soils of plots with different treatments. We hypothesize that this variation in the most biologically active 0–5 cm of soil may form a unique, distinctive pattern/composition that tracks the change in organic matter in the litter manipulation experiment. Furthermore, it was hypothesized that improving litter uptake would provide increased POXC substrates in the soil and increase biological activity. At the same time, persistent withdrawal of organic matter would result in decreased dehydrogenase activity and POXC content.

## 2. Materials and Methods

### 2.1. Study Area

**Figure 1:**
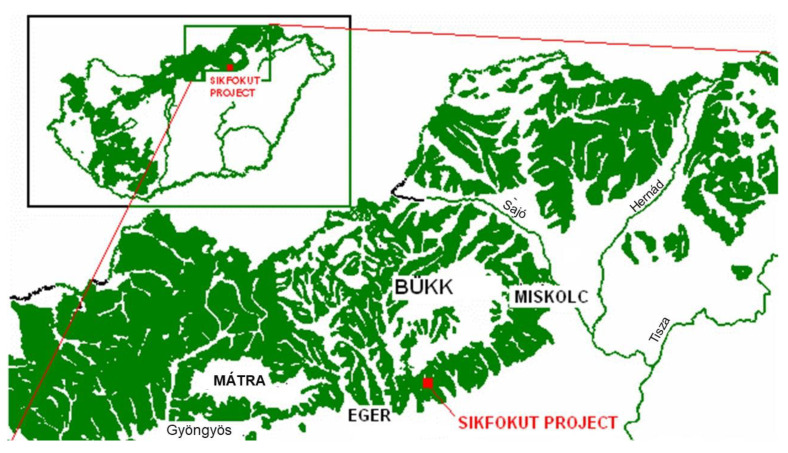
The Síkfőkút DIRT (Detritus Input and Removal Treatment) Project research area in Hungary.

The model area is located in mountains in the north-eastern region of Hungary, 6 km from Eger. GPS coordinates of the Síkfőkút Project: 47°55′; 20°26′, altitude 320–340 m (Figure 1). According to Fekete et al. [8], the mean annual temperature is 10.4 °C and mean annual precipitation is 586 mm. July is the hottest month with an average temperature of 20.2 °C and January is the coldest with −1.2 °C. The 27 ha reserve is vegetated with an even-aged ca. 115-year-old xeric oak forest. The forest has not been managed as an active stand for 115 years. In this previously coppiced even-aged forest, many *Q. petraea* (Matt.) Liebl. and *Q. cerris* L. stems that make up the overstory were 115 years old in 2023. Oak tree species with *Acer campestre* L., *A. tataricum*, *Cerasus avium*, *Carpinus betulus*, and *Cornus mas* comprise the canopy and subcanopy layers. The more important shrub layer species are *A. campestre*, *A. tataricum C. mas*, *Cornus sanguine*, *Crataegus monogyna*, *Euonymus verrucosus*, and *Ligustrum vulgare*. Herbaceous vegetation is significant in the area; a total of 53 species were registered in the vegetation. Detailed description of the vegetation in the area can be found in the works of Koncz et al., Misik et al., and Kotroczó et al. [30,31,32,33].

The soils of the oak forest are Chromic Protovertic Luvisols (Clayic, Cutanic) and Protovertic Endostagnic Abruptic Luvisols (Clayic, Cutanic). The soil was generally characterized by clay loam texture. The clay content was between 30.7% and 46.2% and the silt content was typically between 50.9 and 60.4%. The soil pH: pH_H2O_: was between 6.1 and 5.6 in the 0–5 cm layer of the tested sections [34,35]. Average annual leaf litter production in the area was 3547 kg ha^−1^, while wood debris production was 6230 kg ha^−1^ [33].

The analysis of the decomposition of organic matter in the soil was carried out in the framework of the Síkfőkút DIRT (Detritus Input and Removal Treatment) Project, which is part of the USA ILTER (International Long-Term Ecological Research) DIRT Project. The design of the experimental plots was carried out according to the methods used in the USA DIRT Project. There are three parallel plots per treatment, so a total of 6 × 3 (18) 7 × 7 m experimental plots were created in 2000 (Table 1).

### 2.2. Soil Sampling and Test Methods

Soil samples were collected from the 0–5 cm layer in mineral soil twenty-three years after the treatments were established in the early autumn period. The samples were collected with a 20 mm diameter Pürckhauer 1175/1000 mm soil corer (Bürkle GmbH, Bad Bellingen, Germany).

For the DHA tests, the soil samples were stored at 4 °C after sampling until use. Dry soil samples were used for POXC, SOC, and GRSP analyses. DHA was determined using the reduction of 2.3.5-triphenyltetrazolium chloride (TTC) modified method [36,37]. Samples of 1 g fresh soils were vortexed with 1 mL TTC solution. The samples were incubated at 30 °C. The control contains only 1 mL Tris buffer. After 24 h incubation, triphenyl formazan, a product from the reduction of TTC, was extracted through adding 5 mL methanol into each tube. After shaking, the samples were incubated at room temperature for 2 h in the dark. The soil suspensions (6 mL) were then centrifuged, and the optical density of the clear supernatant was measured against the blank (methanol) at 546 nm.

The amount of soil POXC was determined based on the method of Weil et al. [20]. The well-known name of the method is the determination of permanganate-oxidizable carbon (POXC), which aims to estimate the “active”/”labile” C content of the soil. This carbon source is more accessible to plants and microbes and includes the carbon content stored in the soil’s microbial biomass, organic matter, and carbohydrate molecules. The active organic carbon content that can be determined in this way is more sensitive to the effects of soil interventions than the total organic carbon content. Permanganate-oxidizable carbon was measured with air-dried soil. During this, 10 mL (0.02 M) KMnO_4_ solution was added to 1 g of soil and then shaken for 5 min. After that, the solution was diluted: 200 μL of suspension was added to 10 mL of distilled water. The diluted solution was centrifuged for 5 min, and then the absorbance of the samples was measured at 550 nm. The POXC content is proportional to the depletion of the oxidizing agent, i.e., the fading of the purple colour of potassium permanganate, which results in a lower level of light absorption. The result was quantified based on the method of Blair et al. [19], according to which the consumption of 1 mol of MnO_4_ results in the oxidation of 0.75 mol (9000 mg) of C.

The total carbon content in the soil was determined in triplicate from the homogenized soil samples using a dry combustion method using a Variomax CN Analyzer (ElementARanalySsysteme GmbH, Hanau, Germany). Taking into account the amount of carbon in the carbonates, the samples were heated in a muffle furnace at 450 °C for 16 h and then measured using the combustion method [38].

The Soda Lime (SL) method [39,40] was used to measure soil respiration (CO_2_ emissions). The soda-lime method has been used for a long time in field and laboratory conditions to study soil respiration [41]. We performed two measurements per plots. A known weight of soda lime was placed in two open tins on each plot, and each was covered with a plastic bucket. After 24 h, we collected the tins and measured their mass again under laboratory conditions. The amount of CO_2_ carbon absorbed by soda lime can be calculated from the two dry matter masses (before and after placement) [42].

Extraction of the protein content of soil samples was performed using Khalavati et al.’s [43] method. The essence of the procedure is that 8 mL of 20 mM citrate buffer is added to 2 g of soil sample in autoclavable centrifuge tubes. The samples are then autoclaved for 30 min at 121 °C under high pressure. The resulting solution was centrifuged at 5000 rpm for 15 min, and then the supernatant was used for the tests. The solution contained heat-stable peptides and proteins. The filtrates prepared in this way are analysed using MALDI-TOF MS. 1 µL of sample was dropped onto a slide, and after drying, 1 µL of 70% formic acid was added. Finally, it was sealed with 1 µL α-HCCA (α-cyano-4-hydroxycinnamic acid in 50% acetonitrile and 2.5% trifluoroacetic acid) matrix. The mass spectrum of the preparations was analysed using MALDIT-TOF in the range of 100–1200 Da. A peak list was created from each spectrum, and then these peak lists were averaged per treatment. During the evaluation, we calculated with normalized intensity values, that is, the intensity values belonging to each point were divided back by the intensity value of the base peak. A cluster analysis was then performed on the normalized data.

### 2.3. Statistical Methods

Data processing was performed using IBM Social Science Statistic Packages (SPSS) version 26. DHA, POXc, soil respiration, and SOC concentrations among the treatments were compared using ANOVA and Tukey’s HSD test. Furthermore, we examined the correlation between DHA and POXC. The cluster analysis used to evaluate the soil protein profile was grouped based on the Ward method, and their relationship was represented on a dendrogram, using the squared Euclidean distance. Distance for Ward’s method is:

$$D_{KL}=\frac{||\bar{X_{K}}-{\bar{X_{L}}||}^{2}}{\frac{1}{N_{K}}+\frac{1}{N_{L}}}$$

where ‖…‖^2^ = the Euclidean distance; $\bar{X_{K}}$ and $X_{L}$ = the centroids of clusters *K* and *L*; *N_K_* and *N_L_* are the number of elements in clusters *K* and *L*.

## 3. Results and Discussion

**Figure 2:**
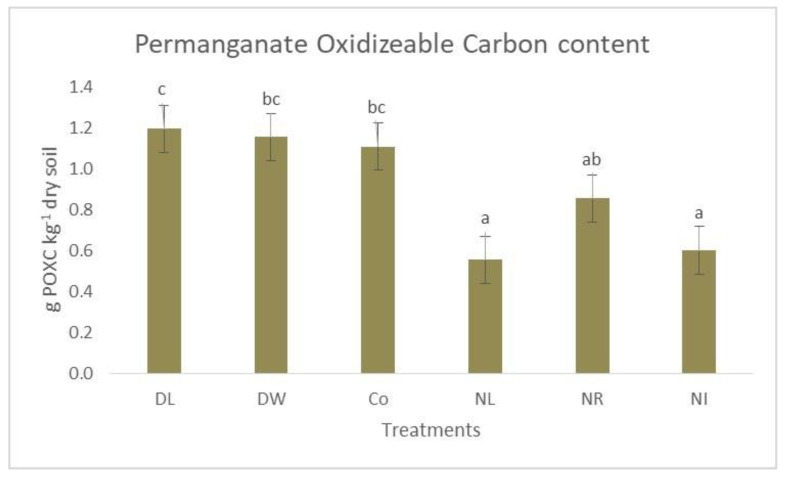
The POXC content of the soil in the 0–5 cm layer of the treatments. The letters represent significant differences between treatments (*n* = 6). Co = Control; DL = Double Litter; NL = No Litter; DW = Double Wood; NR = No Roots; NI = No Inputs.

Based on our results, the Double Litter treatment showed a significantly higher amount of POXC in the upper 5 cm layer of the soil than any litter removal treatment. Similarly, in the case of the Double Wood treatment and the Control plots, we measured higher values than in the case of treatments that did not receive two surface litters (No Litter, No Input) (Figure 2).

**Figure 3:**
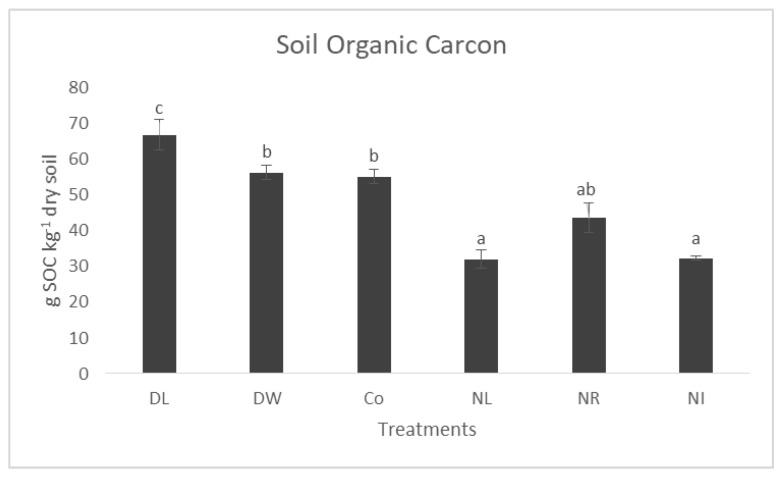
The total organic carbon content of the soil in the 0–5 cm layer of the treatments. The letters represent significant differences between treatments (*n* = 6). Co = Control; DL = Double Litter; NL = No Litter; DW = Double Wood; NR = No Roots; NI = No Inputs.

The differences in the total organic matter content of the soil are also clearly visible 23 years after the treatments were established. In the top 5 cm layer (which means the top 5 cm of the mineral soil under the litter cover), the SOC content of the soils of the Double Litter treatment shows a significantly higher value than that of the litter withdrawal treatments (No Litter, No Roots, No Input) and even the SOC content of the soils of the Control (by 21%) and Double Wood plots. At this depth, the DW and Co treatments differ from the surface litter removal (NL, NI) treatments with significantly higher values (Figure 3).

**Figure 4:**
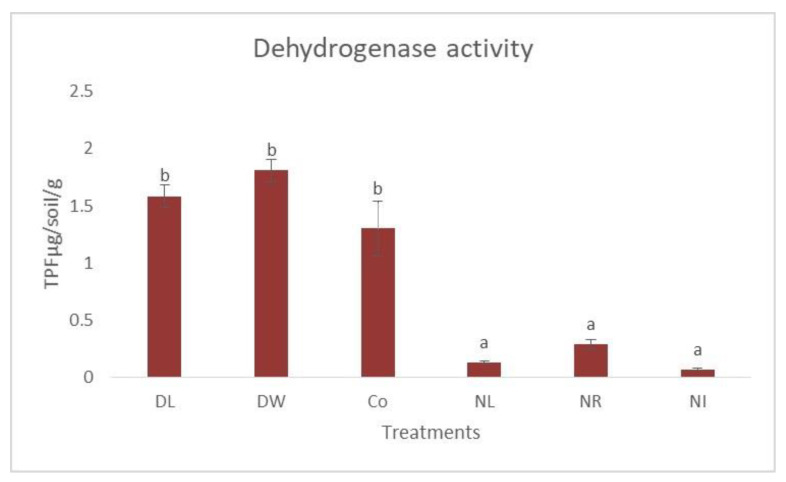
Soil dehydrogenase enzyme activity in the 0–5 cm layer of the treatments. The letters represent significant differences between treatments (*n* = 6). Co = Control; DL = Double Litter; NL = No Litter; DW = Double Wood; NR = No Roots; NI = No Inputs.

In the development of soil dehydrogenase enzyme activity, it is also clearly visible that soil dehydrogenase enzyme activity significantly differed between the litter removal treatments and the Control and doubling treatments (Figure 4).

The results of the soil carbon measurement are shown in Table 2. The data show that the amount of POX carbon compared to the total amount of carbon in the 0–5 cm layer was 1.89% (average of the six treatments). Double Wood and Control treatments showed an above-average value at this depth: Double Wood—2.05%; Control—2.01%. The lowest values for POXC were found in the soils of the treatments not covered by surface soil (No Litter: 1.73%; No Input: 1.81% in the 0–5 cm layer).

This clearly shows the role of surface litter in the development of the amount of labile carbon (POXC). In the soils of the treatments that did not receive surface litter, compared to the control, the amount of POXC decreases more significantly than that of SOC. This can also explain the shift in the ratio mentioned above. Interestingly, in contrast to this trend, the ratio of POXC to the total carbon content was relatively lower in the Double Litter treatment. This is probably due to the fact that the microclimate (the moisture content and temperature do not change as extreme as on the soil without litter covering) is much more balanced under the thick litter cover of the Double Litter treatment [44], so despite the larger amount of fresh litter, due to the more intensive litter decomposition processes, the amount of permanganate-oxidizable carbon content may decrease faster than with other treatments. The sampling took place in November, and the fresh litter that had fallen had not had enough time to get into the soil and start the microbial decomposition processes that contribute to the transformation of organic matter into various forms of carbon. In contrast, a significant part of the amount of labile organic matter associated with last year’s litter fall (or even from previous years) has already decomposed. The results of the autumn soil respiration tests also supported this process.

**Figure 5:**
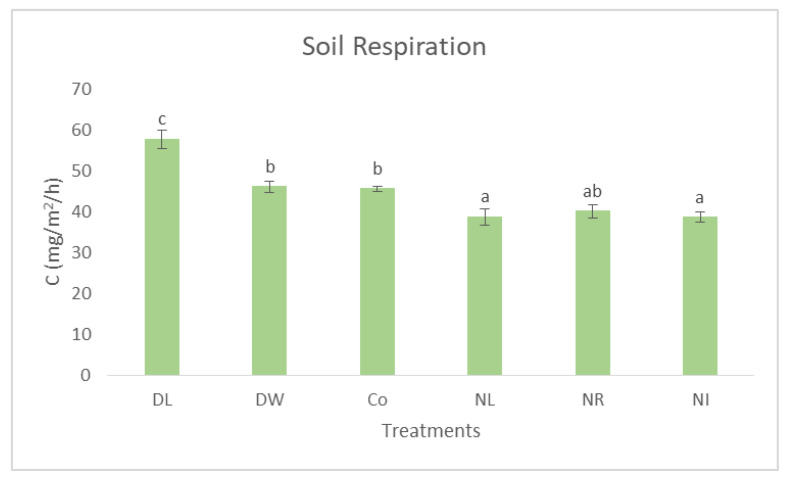
Soil CO_2_ emission in the 0–5 cm layer of the treatments. The letters represent significant differences between treatments (*n* = 6). Co = Control; DL = Double Litter; NL = No Litter; DW = Double Wood; NR = No Roots; NI = No Inputs.

Based on our previous investigations [45] (Fekete et al. 2016), we found that detritus treatments significantly influenced soil microclimate, and thicker litter cover significantly reduced the annual thermal fluctuation of the soil temperature. These differences were significant within the winter (F(6;2191) = 70.31; *p* < 0.01) and the summer periods (F(6;2583) = 65.2; *p* < 0.01). The air temperature was significantly higher in summer than in all soil treatments. Soil temperatures in the root exclusion treatments (NR, NI) were significantly higher than in other soil treatments, and NL was significantly higher than in Co and the litter doubling plots. In contrast, the air temperature was significantly lower than all soil treatments in winter. The aboveground litter exclusion treatments’ (NL and NI) temperatures were significantly lower than the Co and litter addition treatments in winter [42]. Therefore, the DL treatment with a more balanced microclimate showed the highest level of soil respiration, which indicates significantly higher decomposition activity compared to the soils of the other treatments (Figure 5). This process explains the more intense degradation of POXC in DL treatments. Our previous studies also support these results. The amount of fungal biomass (which represents a significant amount of decomposing fungi) was the highest in the Double Litter treatments [46], and the pH values were also the closest to the neutral pH state in the case of the Double Litter treatments [35], and the enzyme activity values also showed this tendency [28,37,42].

**Figure 6:**
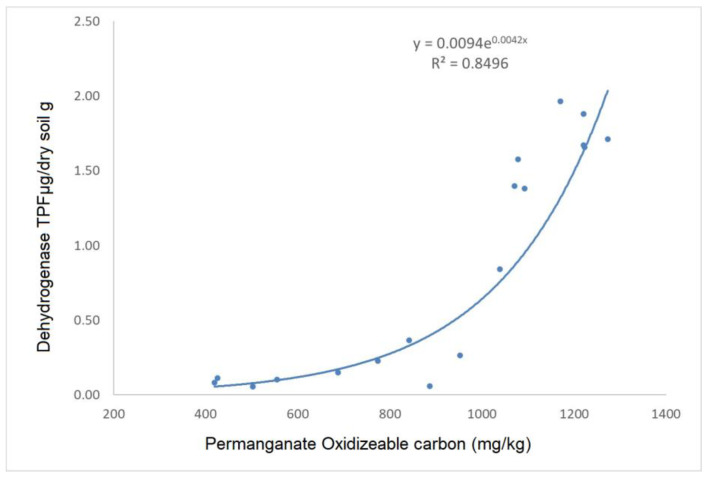
The correlation between the dehydrogenase enzyme activity and the permanganate-oxidizable carbon (POXC) content (r^2^ = 0.85) in the 0–5 cm layer of the treatments.

Since dehydrogenase activity is only performed by living cells, it is a useful indicator of soil microbial activity. Its positive correlation with the POXC content supports the finding that POXC is the fraction of the total carbon content that is most actively used by soil microorganisms. A strong correlation (r^2^ = 0.85) with permanganate-oxidizable carbon showed the connections of POXC with enzyme activity (Figure 6).

The POXC stock is particularly sensitive to microclimatic changes. As a result, they can change their amount in soils even in the short term [47]. This feature strengthens their indicator character. SOC accumulation and soil respiration are interdependent, parallel processes. In these processes, environmental factors (mostly the climate, the quality parameters of the organic compounds involved, and the physical-chemical nature of the soil environment) control whether soil respiration or changes in the amount of SOC will be the dominant response to decreases or increases in litter intake. [8,42].

**Figure 7:**
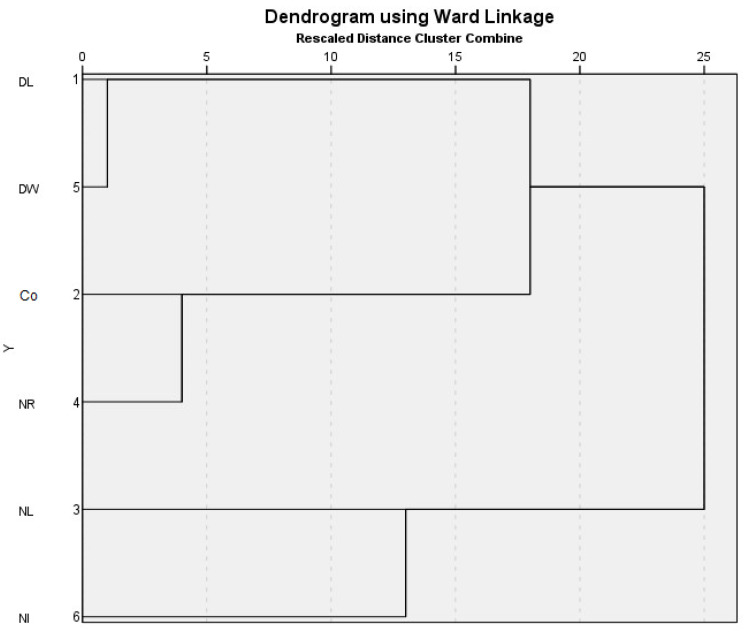
Dendrogram showing the results of the cluster analysis grouping of heat stable protein sequence in the 0–5 cm layer of the treatments. X-axis distance is based on the Squared Euclidean Distance. Co = Control; DL = Double Litter; NL = No Litter; DW = Double Wood; NR = No Roots; NI = No Inputs.

During our tests, we also analysed the protein pattern of the soil [48]. We collected 10,640 peaks per sample in the investigated mass range. Based on the dendrogram of the cluster analysis of the data (Figure 7), the treatments can be separated into two main groups. It can be concluded that the NI and NL treatments were significantly different from the other litter treatments and from the Control. The spectra of the DL and DW treatments and of the Co and NR treatments showed close similarity. These trends overlap with the POXC results indicating microbial activity. The protein patterns of the soils of the treatments receiving surface litter are closely related to each other and are significantly different from the litter withdrawal treatments.

Knicker and Hatcher [49] and Zang et al. [50] reported that proteins are incorporated into the hydrophobic regions of soil organic matter, thereby partially protecting them against degradation processes. However, when analysing soil proteins and their carbon content, He et al. [23] found that AMF mycorrhizal treatment of soils had a significantly greater contribution to the SOC content of soils than soils without GRSP. It is also clear from our results that SOC content is higher in the treatments that receive surface litter, and this is proportional to the protein content of the soil, which is also a significant carbon store in soil [51]. Carbon stored in GRSP is extremely resistant (lasts 12–22 years) [52,53] and mostly plays an active role in the formation of soil structure [54], but some of its fractions contribute through microbial degradation processes to soil nutrient supply processes [55]. Furthermore, GRSP protects the soil’s carbon reserves, including POXC, which helps regulate the nutrient supply of plants [53,56].

The soils of the different treatments reacted differently to removing or doubling organic matter. After 23 years, the withdrawal of litter and the accompanying decrease in organic matter greatly affected the enzyme activity values compared to increased litter input. In the case of soil dehydrogenase activity, the withdrawal of litter significantly reduced enzyme activity; however, doubling the amount of litter did not significantly increase enzyme activity. The permanganate-oxidizable carbon content of the soil can be easily used by the microbes living in the soil. Similar to biological activity, in the long term, the deprivation treatments differed significantly from the Control treatment, while similar results were obtained in the case of the organic matter doubling treatments. These relationships developed similarly at other DIRT research sites. At the temperate deciduous DIRT research site (Harvard Experimental Forest, MA, USA), C concentrations decreased during the first decade of experimental litter reduction [17] and were even more pronounced after twenty years [57]. Similar soil C reductions were reported after studies at two other DIRT experimental sites and also in temperate forests (Bousson Environmental Research Reserve, Allegheny College and the University of Wisconsin Arboretum (USA)) [58,59] (Bowden et al., 2014; Lajtha et al., 2014).

## 4. Conclusions

Investigating the processes that influence the degradation and accumulation of SOC in soils helps to understand and predict how changes of different origins shape organic matter and, in part, the nutrient storage capacity of soils, in addition to the extent to which organic carbon stored in soils is actively involved in the carbon cycle and how quickly it is returned to the atmosphere. Climate change influences soil temperature and moisture content directly through the change of litter production. Changes in vegetation also have an impact on litter production. Its reduction can impede nutrient recycling and, through the thinning of the litter cover, alter the microclimate of soils, which become warmer in summer and colder in winter.

In contrast, an increase in litter production dampens the effects of weather extremes, reflected in the number of days with frosty and warmer than 20 °C average temperatures. As a result of favourable microclimatic conditions, POXC decomposes faster in the soils of the DL treatment than in the control or DW soils; on the other hand, the total amount of SOC increases in this treatment. This shows that in the climate typical of the DIRT research area in Síkfőkút, growing leaf litter can increase the carbon content of soils even with more intensive decomposition processes. Our results demonstrate the importance of soil organic matter, both through microclimatic factors and biological processes in the soil, over the long term.

Testing the protein pattern of soils is a new possibility. Since proteins are primarily related to microbes and their activities, the diversity of proteins reflects the microbiome’s composition. Our study focused on heat-stable proteins, thereby narrowing down the wide range of proteins in soils. The cluster analysis results of the data showed similarities with the results of enzyme and POXC measurements. Since these mainly characterize the entire activity of the soil, the protein pattern can also be characteristic of the entire microbiome. The vegetation of the experimental site is zonal oak forest, where a large part of the soil microbiome is made up of filamentous fungi, so the question is how many of the tested thermostable proteins in the GRSP are part of the soil’s organic carbon pool. If, in the future, litter production and the amount of organic matter in the soil will decrease, this will have an impact on decomposition processes in the soil and, more broadly, on nutrient cycling, as well as on SOC dynamics, soil CO_2_ emission, biological activity, and microbiome composition. If litter production were to increase, increased organic matter inputs would not cause such rapid and significant changes in soil processes as a decrease in litter production.

## Figures and Tables

**Table 1:** The DIRT (*Detritus Input and Removal Treatments*) treatments at Síkfőkút Project site (Hungary).

Applied Treatments	Description of Treatments
Control (Co)	Average litter amount typical of the forest site.
No Litter (NL)	Above-ground inputs are excluded from plots. Leaf litter was removed using a rake. This process was repeated continuously every year.
No Roots (NR)	The plots were trenched around 40 cm wide and 100 cm deep. The soil dug out was placed outside the plot. Root-proof Delta MS 500 PE foil, which was 0.6 mm thick and 1 m wide and of high density, was put in the trenches. Then, the trenches were filled with soil. So as to eliminate root production, plants were cleared (bushes had been cut out at the establishment).
No Inputs (NI)	Aboveground inputs are excluded from plots; the belowground inputs are provided as in NR plots. This treatment is the combination of NR + NL plots.
Double Wood (DW)	Aboveground wood debris inputs are doubled through adding wood to each plot. Annual wood litter amount was measured using boxes placed at the site, and its double amount was applied in the case of every DW plots.
Double Litter (DL)	Above-ground leaf inputs are doubled through adding litter removed from No Litter plots.

**Table 2:** Evolution of soil organic carbon content (SOC) and permanganate-oxidizable carbon content (POXC).

Treatments	Total-C (g SOC kg^−1^ Dry Soil)	POX-C (g POXC kg^−1^ Dry Soil)
Double Litter (DL)	66.9	1.2
Double Wood (DW)	56.3	1.2
Control (Co)	55.2	1.1
No Litter (NL	32.1	0.6
No Roots (NR)	43.6	0.9
No Inputs (NI)	32.3	0.6

## Data Availability

Data are contained within the article.
